# Bringing online adaptive radiotherapy to a standard C-arm linac

**DOI:** 10.1016/j.phro.2024.100597

**Published:** 2024-06-07

**Authors:** Maureen L. Groot Koerkamp, Gijsbert H. Bol, Petra S. Kroon, Lean L. Krikke, Tessa Harderwijk, Annelies J. Zoetelief, Annick Scheeren, Stefan van der Vegt, Annika Plat, Jochem Hes, Ineke B.A. van Gasteren, Esmee R.T. Renders, Reijer H.A. Rutgers, Saskia W. Kok, Joost van Kaam, Geja J. Schimmel-de Kogel, Gonda G. Sikkes, Dennis Winkel, Michael J. van Rijssel, André J.M. Wopereis, Kübra Ishakoglu, Juus L. Noteboom, Jochem R.N. van der Voort van Zyp, Naomi Beck, Timo F.W. Soeterik, Sandrine M.G. van de Pol, Wietse S.C. Eppinga, Corine A. van Es, Bas W. Raaymakers

**Affiliations:** Department of Radiotherapy, UMC Utrecht, Heidelberglaan 100, 3584CX Utrecht, the Netherlands

**Keywords:** Online adaptive radiotherapy (oART), Image-guided radiotherapy, Cone-beam computed tomography, Linear accelerator, Bladder cancer

## Abstract

Current online adaptive radiotherapy (oART) workflows require dedicated equipment. Our aim was to develop and implement an oART workflow for a C-arm linac which can be performed using standard clinically available tools. A workflow was successfully developed and implemented. Three patients receiving palliative radiotherapy for bladder cancer were treated, with 33 of 35 total fractions being delivered with the cone-beam computed tomography (CBCT)-guided oART workflow. Average oART fraction duration was 24 min from start of CBCT acquisition to end of beam on. This work shows how oART could be performed without dedicated equipment, broadening oART availability for application at existing treatment machines.

## Highlights

• We developed an online adaptive radiotherapy (oART) workflow.
• The oART workflow is cone-beam computed tomography-guided.
• The oART workflow can be performed on a standard C-arm linac.
• The first patients with bladder cancer were successfully treated with the workflow.

## 1 Introduction

Interfraction motion is one of the major challenges in radiotherapy. The bladder is a site in which potentially large interfraction motion has to be considered, caused by differences in bladder filling, rectum filling and bowel position [1], [2], [3]. In non-adaptive workflows, large planning target volume (PTV) margins are required to take into account the differences. A plan library approach could be used to deal with interfraction variation while using smaller margins [4], [5]. With online adaptive radiotherapy (oART) the radiotherapy plan can be adapted on daily imaging to correct for interfraction anatomical changes [6]. However, oART has to be fast to outperform a plan library [7]. Several planning studies have shown that oART with plan reoptimization has the potential to use smaller margins and better spare adjacent tissue [8], [9], [10], [11].

Currently, all available oART workflows require dedicated equipment, such as an magnetic resonance (MR)-Linac [12], [13] or dedicated cone-beam computed tomography (CBCT)-guided machine [14]. To increase oART availability, our goal was to develop, clinically implement, and evaluate an oART workflow that can be performed without dedicated equipment on a standard C-arm linac with requirements to use clinically approved tools, to minimize user interaction, and to be fast, using the bladder as target site for this proof-of-concept.

## 2 Materials and methods

### 2.1 Workflow design

**Fig. 1:**
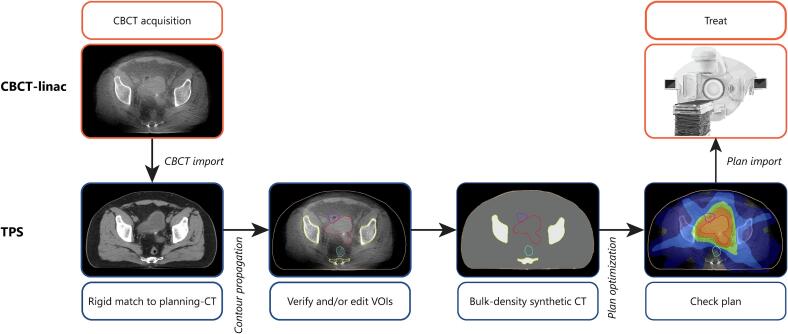
The online adaptive CBCT-guided radiotherapy workflow design. The overview shows the different steps and tools used in the online adaptive radiotherapy (oART) workflow for a palliative radiotherapy treatment of a patient with bladder cancer. The contours show the bladder CTV (red), oART PTV (green), bowel bag (purple), rectum (cyan), bony structures (yellow), and body (beige). The orange isodose line represents 95% of the prescribed dose.

The oART workflow (Fig. 1) was developed with clinically existing tools: XVI (Elekta AB, v5.0.4. and v5.0.7.) for CBCT acquisition, Monaco treatment planning system (TPS) including Monaco scripting (Elekta AB, v6.00.01) for recontouring and plan optimization, and Mosaiq (Elekta AB, v2.83) as treatment delivery software. Treatment delivery was performed on an Elekta Synergy C-arm linac. The daily CBCT (Supplemental Table S1) was sent to the TPS. With the TPS tools, planning-CT contours were propagated to the CBCT with rigid (body) or deformable image registration (other contours). Bulk-densities were assigned to body (water density), femurs and remaining bony structure contours (average planning-CT density). For speed and safety, the TPS workflow part was scripted using Monaco scripting and user interaction was only required for patient selection, contour editing, and plan approval. The PTV was recreated after CTV approval. A single 360° volumetric modulated arc therapy plan was optimized from scratch without user interaction with a generic planning template. A 4x4x4 mm^3^ calculation grid size and reduced number of iterations were used to accelerate optimization. The TPS multicriterial optimization option was used to optimize organ at risk (OAR) sparing. We applied a dose volume histogram (DVH) parameter score card for PTV V95% and V107%, and dose fall-off at 2 cm (D0.1cc) and 4 cm (D1cc) from the PTV to facilitate quick online plan evaluation. After export from the TPS, manual import of the plan in Mosaiq was required. In-house developed tools were used to send the data between the different systems and to add safety measures to minimize the risks identified in a risk analysis.

### 2.2 Risk analysis

To identify risks and implement mitigation strategies for clinical implementation of the workflow, we performed an extensive risk analysis of steps differing from the conventional image-guided radiotherapy workflow. The analysis was performed by a multidisciplinary team using the Healthcare Failure Mode and Effects Analysis (HFMEA) method [15].

### 2.3 Workflow testing

Automated planning template performance was tested on ten retrospective patients (informed consent waived for use of retrospective data, approval number WAG/mb/20/500028) for two CBCTs per patient and compared to recalculation of manually generated clinical plans on the same anatomy. The plans were compared on plan parameters (monitor units (MU), optimization and estimated delivery time) and DVH parameters (clinical target volume (CTV) coverage, dose to OAR). Additionally, multiple workflow tests were performed to test the workflow and safety mitigations with retrospective patient data or the Alderson RANDO phantom (Radiology Support Devices). As final check before clinical introduction, two end-to-end tests with each two fractions were performed. Finally, quality assurance (QA) measurements with a Delta4 phantom (ScandiDos AB) were used to test five retrospective test plans and nine plans generated during workflow tests. These were evaluated using 3% dose deviation/3mm distance-to-agreement criteria with a 95 % gamma index threshold.

### 2.4 First treatments

Three patients with an indication for palliative bladder radiotherapy (10–13 fractions of 3 Gy) were treated with the workflow, which was implemented as approved clinical procedure. Informed consent was obtained for published images. Per patient two plans (Table S3) were prepared on a planning-CT with empty (patient 1 and 3; empty bladder 30 min before treatment, no water intake) or medium bladder filling (patient 2; empty bladder and drink 200 cm^3^ water 45 min before treatment): a reference plan for the oART workflow and a back-up plan created according to the non-oART current clinical practice. PTV margins applied were: 1.5 cm to superior/anterior/posterior and 1.0 cm to left/right/inferior for the back-up plan, and 1.6 cm to superior, 1.5 cm to anterior, 1.0 cm to left/right/posterior, and 0.6 cm to inferior for the oART plan. The oART PTV margin was based on a literature study considering an intrafraction duration of 25 min [7], [16], [17], [18], [19]. A post-CBCT was acquired in the first three oART fractions, and if required one extra fraction, to check if the CTV remained within the PTV. Planning constraints for both plans were: PTV V95% ≥ 95% (acceptable) and ≥ 99% (preferred), and Dmax < 107% of prescribed dose, OAR dose as low as reasonably achievable.

Each oART fraction was performed by three radiotherapy technicians trained for the workflow. Additionally present were a radiation oncologist for supervision of contouring and plan approval, and a technical physician for assistance with the oART workflow. A medical physicist was available on call.

We evaluated the number of fractions delivered with the oART workflow, duration of oART workflow steps, number of MU of the plans, QA measurements of the oART reference plan and the first two adaptive plans of each patient, CBCT image quality (subjective user feedback), and CTV and PTV volumes. Additionally, we compared dosimetric parameters (CTV V95%, OAR Dmean) of the oART plans to forward recalculation of the back-up plan as simulation of the conventional workflow.

Analyses were performed using descriptive statistics (Rstudio v2022.12.0).

## 3 Results

The risks with highest risk scores were incorrect bulk-density assignment, insufficient CBCT quality and incorrect PTV generation (Table 1).

**Table 1:** Biggest risks identified in the HFMEA risk analysis and corresponding mitigation strategies. These are the risks scored with a ‘Catastrophic’ (Ca) severity or ‘High’ (H) or ‘Very high’ (VH) hazard score. ED = electron density; TPS = treatment planning system.

Risk	Risk scoring	Mitigation strategy
Incorrect bulk-density settings	Ca; VH	1. Check in TPS script: to check if ‘force ED’ setting is applied to structures on the reference CT for the contours that require a bulk-density assignment. 2. Check in TPS script: to check if the ‘fill ED’ setting is not applied to any structure.
Insufficient CBCT quality for online contouring	H	Switch to back-up workflow if CBCT quality is insufficient for online contouring.
Failure of ‘Recreate margins’ option required for PTV update	Ca	Prevent manual editing of the propagated PTV contour, which causes failure, by training of staff involved and two pairs of eyes present when editing contours. Switch to back-up workflow if PTV contour is accidentally edited manually.
Incorrect PTV margin creation with slice thickness differences between planning-CT (3 mm) and CBCT (2 mm)	H	Set PTV margin in TPS to 16 mm cranially (instead of 15 mm) and 6 mm caudally (instead of 5 mm) to account for rounding differences between 2 mm and 3 mm slice thickness in cranial and caudal direction.
CBCT is matched to planning-CT before exporting to TPS (=wrong coordinate system)	Ca	In-house filter to check for correct export option set in CBCT DICOM before importing CBCT in TPS.
Incorrect CBCT orientation	Ca	In-house filter to check orientation in CBCT DICOM before importing CBCT in TPS.
Wrong CBCT exported	Ca	In-house filter to check CBCT acquisition date before importing CBCT in TPS.
Change in table position after CBCT acquisition	Ca	Table management software is set to allow only a table position change before CBCT acquisition.
Plan isocenter incorrectly defined	Ca	In-house filter to check if isocenter coordinates are equal to (0,0,0) at plan export.
Reference plan selected for irradiation instead of the online adaptive plan	Ca	1. In-house filter to set reference plan pedestal position to 90 degrees to trigger a warning based on incorrect table position when this plan is accidentally selected. 2. In-house filter to set reference plan to 1 MU in the treatment delivery software, such that when all warnings are overridden not more than 1 MU can be irradiated.

The automated planning template tests showed good plan quality compared to the manually created plans with a 99% median PTV coverage for both plans, a 4 Gy median lower rectum Dmean, but an increased median number of MU of 1077 vs. 620 MU (Table S2) for the oART plans. Median gamma pass rate was 99.8%.

Thirty-three of 35 fractions were successfully delivered with the oART workflow. The back-up workflow was used in one fraction for the second and the third patient, because of a failure in the scripted TPS workflow part and a TPS shutdown respectively. Average fraction duration was 24 min from start of CBCT acquisition to end of beam on and contour editing required the most time (Supplemental Fig. S1). The automated plan optimization for the oART plans resulted in higher MU than manual the back-up plans: median 1086 vs. 764 MU respectively. Median gamma pass rate was 100%.

CBCT image quality was acceptable for contouring in all fractions, though CBCTs of three fractions in the first patient were severely impacted by artefacts caused by air in bowel or rectum. Additionally, the low amount of fatty tissue between organs complicated contouring in this patient. Imaging software version 5.0.7. (third patient) showed reduced saturation towards the body contour compared to version 5.0.4. (first and second patient).

With a range of 178 cm^3^, CTV volume across fractions differed most for the second patient (Supplemental Fig. S2). The CTV extended the PTV cranially in the fraction 1 post-CBCT for this patient. We changed bladder filling instructions for this patient from fraction three onwards to empty bladder to stabilize bladder filling. The CTV remained within the PTV on the other post-CBCTs and for the other patients. Median PTV volume was 717 cm^3^ for the oART plans compared to 663 cm^3^ for the back-up plans.

The dose/volume based evaluation of oART plans vs. forward calculation of the back-up plan showed a median CTV V95% of 100% for both approaches, a median 4.6 Gy lower rectum Dmean and 0.8 Gy bowel bag Dmean for the oART approach (Supplemental Fig. S3). Visual inspection of the CBCTs indicated that a bladder volume smaller than on the planning-CT usually resulted in a lower bowel bag dose, whereas a larger volume resulted in a higher bowel bag dose compared to the back-up plan (Supplemental Fig. S2).

## 4 Discussion

This work described the development and clinical implementation of a CBCT-guided oART workflow on a standard C-arm linac and treatment of the first three patients. Workflow automation for speed and safety purposes was achieved by using TPS scripting from contour propagation to plan export. This work shows that oART is possible with currently clinically available tools without dedicated equipment. The first patients were treated successfully.

Several dedicated oART solutions are available [12], [13], [14]. This work shows that oART is possible without the need for dedicated machinery. Previously, Wong et al. have already shown that a similar concept could be implemented for a one-step ‘simulate-and-treat process’ using simple beam geometry [20]. The current work shows that the approach could be translated to a daily oART setting with reduction in time required for treatment planning and plan approval while applying more complex treatment planning, though automated plan optimization resulted in a high number of MUs. Use of standard equipment may increase the availability of oART, also for departments without dedicated oART machines or MRI experience, and allows more flexibility in treatment time and hardware allocated for oART.

With the dedicated systems, oART has also been applied for treatment of bladder cancer [16], [17], [18], [19], [21], [22]. This study showed shorter treatment durations compared to 30–40 min in MR-Linac reports [16], [18] and durations comparable to CBCT-guided experiences [17], [19]. Similar to our observations for the rectum, other studies reported improved sparing of rectum and bowel with the use of smaller PTV margins in the oART workflow [17], [19]. However, to extend application of the workflow to curative bladder patients, at our department the oART workflow would have to compete to a plan library approach. Based on the findings of Den Boer et al., an oART workflow for bladder should be performed in approximately 15 min to outperform a library of plans approach in terms of healthy tissue volume inside the PTV and target coverage [7]. Currently, this oART workflow is not fast enough to reach this.

Workflow duration also has to reduce for oART to become a significant alternative to non-adaptive image-guided radiotherapy for other indications and lengthier treatment schedules. Based on the current timings, the largest gains could be made in reduction of time required for contouring and plan optimization. Imaging duration at the C-arm linac was limited by the maximum rotation speed of 360 degrees/min. Improvement of CBCT image quality will help user-based contouring but may also improve contour propagation results. Additionally, alternative contour propagation methods such as anatomically-adaptive deformable registration or patient-specific neural networks may help in reducing contouring time [23], [24]. Plan optimization time may be reduced implementing GPU-based calculation methods [25], [26]. Our largely scripted implementation of the workflow and automated treatment planning ensured reproducibility and safety, but also served speed requirements.

Though the workflow shows how oART can be performed on a standard CBCT-guided system, it required implementation of additional risk mitigation checks between different software components. This may limit wider implementation for other treatment sites and in other centers. Better integration of different workflow parts would mitigate the need for home-built safety measures, improve usability of the workflow and workflow speed.

In conclusion, this work described the development and clinical implementation of a CBCT-guided oART workflow that can be performed without dedicated equipment. The first clinical experiences show the successful application of the workflow for palliative radiotherapy treatment of patients with bladder cancer.

## CRediT authorship contribution statement

**Maureen L. Groot Koerkamp:** Conceptualization, Methodology, Validation, Formal analysis, Investigation, Writing – original draft, Writing – review & editing, Visualization. **Gijsbert H. Bol:** Conceptualization, Methodology, Software, Validation, Investigation, Resources, Writing – review & editing. **Petra S. Kroon:** Conceptualization, Methodology, Validation, Investigation, Writing – review & editing, Supervision. **Lean L. Krikke:** Methodology, Validation, Investigation, Writing – review & editing. **Tessa Harderwijk:** Methodology, Validation, Investigation, Writing – review & editing. **Annelies J. Zoetelief:** Methodology, Validation, Investigation. **Annick Scheeren:** Methodology, Validation, Investigation. **Stefan van der Vegt:** Methodology, Validation, Investigation, Writing – review & editing. **Annika Plat:** Methodology, Validation, Investigation, Writing – review & editing. **Jochem Hes:** Methodology, Validation, Investigation, Writing – review & editing. **Ineke B.A. van Gasteren:** Investigation, Resources, Writing – review & editing. **Esmee R.T. Renders:** Investigation, Writing – review & editing. **Reijer H.A. Rutgers:** Investigation, Writing – review & editing. **Saskia W. Kok:** Investigation, Writing – review & editing. **Joost van Kaam:** Investigation, Writing – review & editing. **Geja J. Schimmel-de Kogel:** Methodology, Writing – review & editing. **Gonda G. Sikkes:** Methodology, Writing – review & editing. **Dennis Winkel:** Methodology, Software, Investigation. **Michael J. van Rijssel:** Validation, Writing – review & editing. **André J.M. Wopereis:** Validation, Resources, Writing – review & editing. **Kübra Ishakoglu:** Validation, Resources, Writing – review & editing. **Juus L. Noteboom:** Writing – review & editing, Supervision. **Jochem R.N. van der Voort van Zyp:** Writing – review & editing, Supervision. **Naomi Beck:** Investigation, Writing – review & editing. **Timo F.W. Soeterik:** Investigation, Writing – review & editing. **Sandrine M.G. van de Pol:** Investigation, Resources, Writing – review & editing, Supervision. **Wietse S.C. Eppinga:** Investigation, Resources, Writing – review & editing, Supervision. **Corine A. van Es:** Investigation, Resources, Writing – review & editing, Supervision. **Bas W. Raaymakers:** Conceptualization, Methodology, Writing – review & editing, Supervision, Funding acquisition.

## Declaration of Competing Interest

The authors declare the following financial interests/personal relationships which may be considered as potential competing interests: Our department has a research agreement with Elekta AB on the development of adaptive strategies for the CBCT-linac. The first author is working on a grant for this purpose, which is partly financed by Elekta AB. Elekta AB had no role in the preparation, review, or approval of the manuscript and the decision to submit the manuscript.
