# Supplementary material for: Bringing online adaptive radiotherapy to a standard C-arm linac
Source: Phys Imaging Radiat Oncol. 2024 Jun 7;31:100597. doi: 10.1016/j.phro.2024.100597 (PMC11239695; doi:10.1016/j.phro.2024.100597)
Supplement: Supplementary Data 1 [file mmc1.pdf]

Supplementary material

Table S1. CBCT protocol settings.

**Peak kilo voltage (kV)**	120
**Exposure time / Nominal ms per frame (ms)**	40
**X-Ray tube current / Nominal mA per frame (mA)**	64
**Filter**	Bowtie filter
**Gantry rotation speed (degrees/min)**	270 / 360
**Field-of-view (cm)**	41x41x26.4
**Reconstruction voxel size (mm)**	1x1x1

Table S2. Results of the automated planning template performance compared to the clinically used treatment plans for ten retrospective patients for two CBCTs each. Mean dose (Dmean) difference is calculated as: oART Dmean – recalculation of clinical plan Dmean.

	**oART template**<br>*Median [range]*	**Clinical plan**<br>*Median [range]*
**PTV V95%**	99.3% [97.4-99.8%]	99.0% [98.3-99.2%]
**Rectum Dmean difference**	-4.3 Gy [-11.8-3.2 Gy]	
**Bowel bag Dmean difference**	-0.9 Gy [-4.4-5.1 Gy]	
**Plan optimization time**	2 min 31 s<br>[1 min 39 s - 4 min 18 s]	-
**Number of MU**	1077 [827-1301]	620 [536-814]
**Expected delivery time**	139 s [110-160 s]	102 s [98-128 s]

Table S3. Radiotherapy plan template settings.

	**oART template**	**Back-up plan template**
**Number of arcs**	1x 360°	1x 360°
**Grid spacing (cm)**	0.4	0.3
**Statistical uncertainty (per control point)**	8%	8%
**Fluence smoothing**	Medium	Medium
**Maximum segments**	144	144
**Minimum segment width (cm)**	0.5	1.0
**SSO loops**	5	14
**IMRT constraints**	PTV<br>Rectum (MCO)<br>Bowel (MCO)<br>body	PTV<br>Rectum<br>Bowel<br>body
**Bulk-density contours**	Body (RED = 1.000)<br>Bony structures (RED = average from planning-CT<br>Femur_L and Femur_R (RED = average from planning-CT)	None*<br>**Bowel and rectum: fill to RED = 1.000 (all voxels <1.000 are set to 1.000)*

MCO = multicriterial optimization
RED = relative electron density
SSO = segment shape optimization

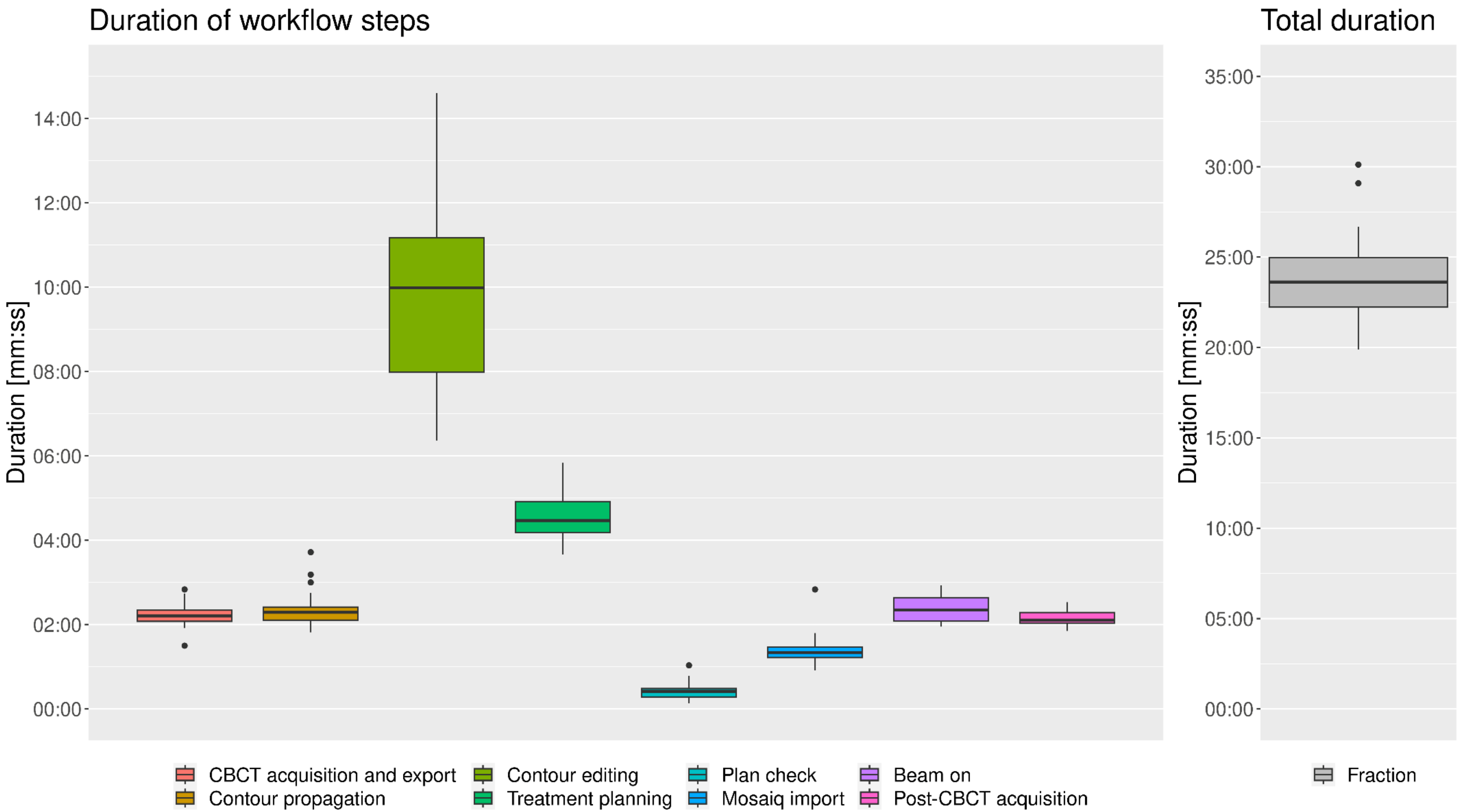

Figure S1. Duration of the different steps of the oART workflow and the total duration of a fraction (from start of CBCT acquisition to end of beam on) for 33 oART fractions of palliative-intent radiotherapy in three patients with bladder cancer. The treatment planning step included couch import, plan template import and a full plan optimization from scratch.

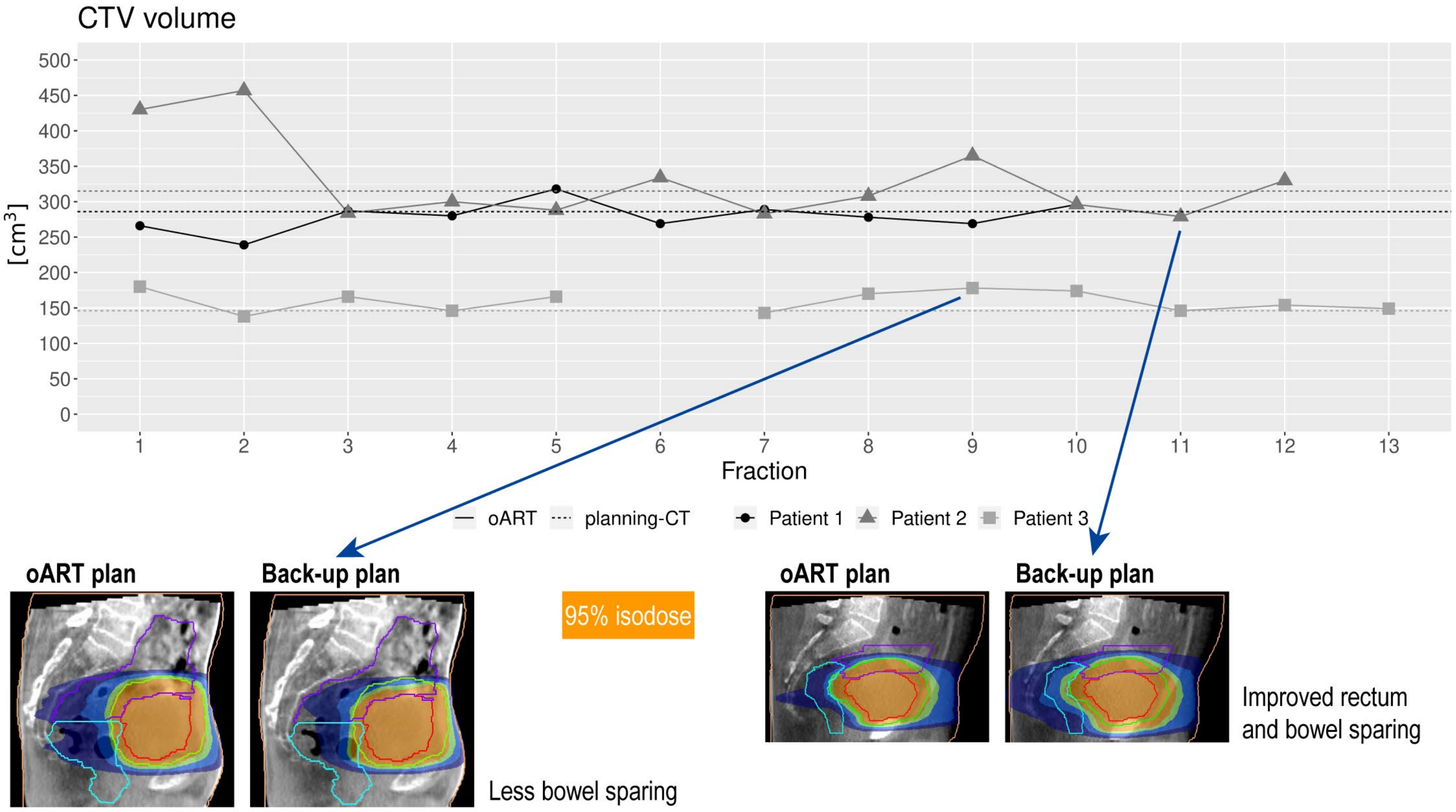

Figure S2. Comparison of oART CTV volumes and planning-CT CTV volume. At the bottom two examples show the differences in dose distributions between the oART plan and recalculation of the back-up plan on the CBCT anatomy showing the effect of the CTV (red contour) volume changes on dose to the rectum (cyan) and bowel bag (purple).

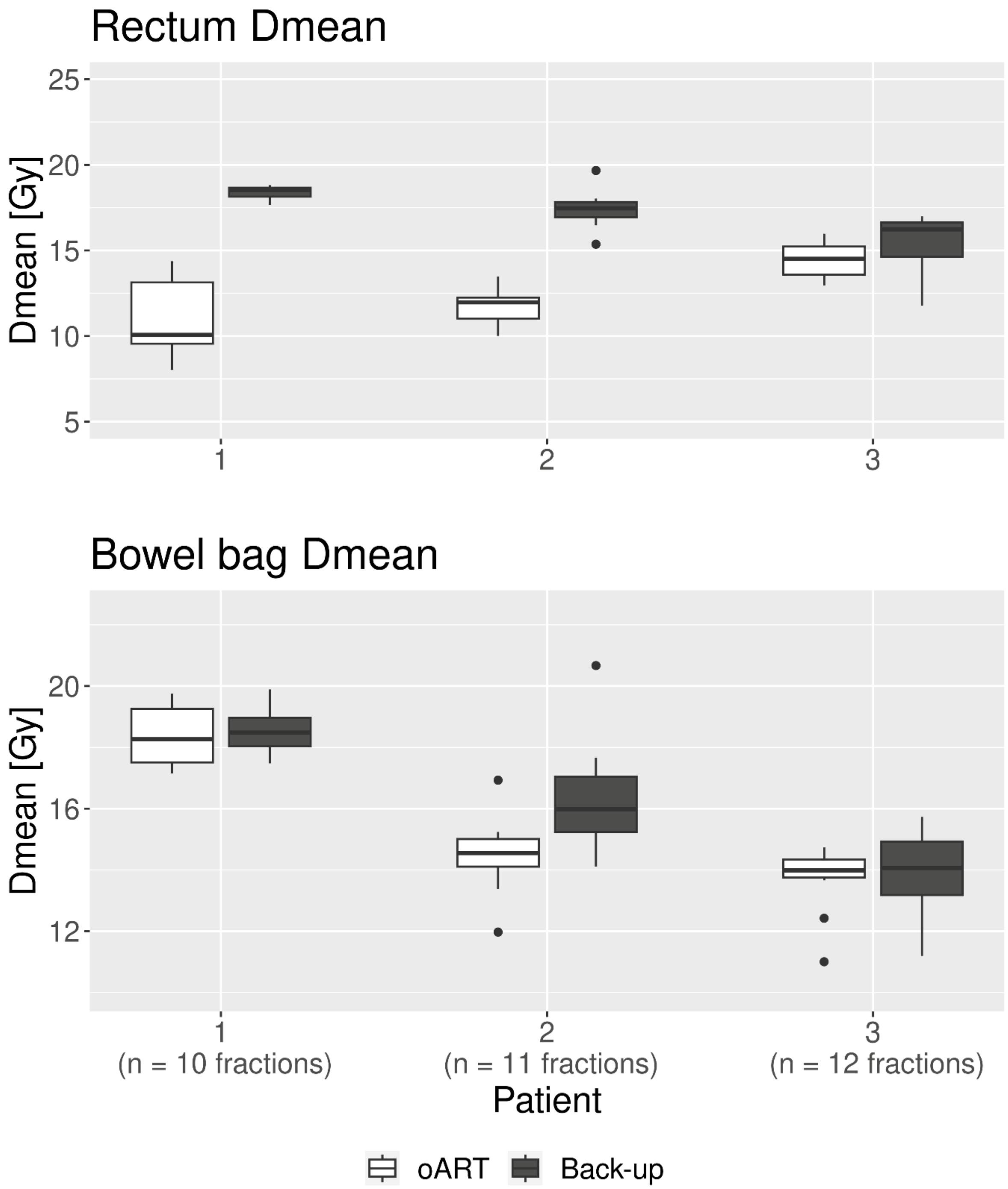

Figure S3. Comparison of mean dose to rectum and bowel bag between oART plans and forward calculation of the back-up plan on the same anatomy. Reference prescribed total dose for plan optimization: 30 Gy for patient 1 and 2, 39 Gy for patient 3. Actual treated dose: 30 Gy (10 oART fractions) for patient 1, 36 Gy (11 oART fractions, 1 back-up fraction) for patient 2, 39 Gy (12 oART fractions, 1 back-up fraction) for patient 3.
